# Geo-Positioning Accuracy Improvement of Multi-Mode GF-3 Satellite SAR Imagery Based on Error Sources Analysis

**DOI:** 10.3390/s18072333

**Published:** 2018-07-18

**Authors:** Niangang Jiao, Feng Wang, Hongjian You, Xiaolan Qiu, Mudan Yang

**Affiliations:** 1 Key Laboratory of Technology in Geo-Spatial Information Processing and Application Systems, Institute of Electronics, Chinese Academy of Sciences, Beijing 100190, China; fwang2@mail.ie.ac.cn (F.W.); hjyuo@mail.ie.ac.cn (H.Y.); xlqiu@mail.ie.ac.cn (X.Q.); mdyang1993@163.com (M.Y.); 2 Institute of Electronics, Chinese Academy of Sciences, Beijing 100190, China; 3 School of Electronic, Electrical and Communication Engineering, University of Chinese Academy of Sciences, Beijing 100049, China

**Keywords:** multi-mode GF-3 satellite images, geometric performance, block adjustment, rational function model (RFM), preconditioned conjugate gradient (PCG) algorithm, error sources analysis.

## Abstract

The GaoFen-3 (GF-3) satellite is the only synthetic aperture radar (SAR) satellite in the High-Resolution Earth Observation System Project, which is the first C-band full-polarization SAR satellite in China. In this paper, we proposed some error sources-based weight strategies to improve the geometric performance of multi-mode GF-3 satellite SAR images without using ground control points (GCPs). To get enough tie points, a robust SAR image registration method and the SAR-features from accelerated segment test (SAR-FAST) method is used to achieve the image registration and tie point extraction. Then, the original position of these tie points in object-space is calculated with the help of the space intersection method. With the dataset clustered by the density-based spatial clustering of applications with noise (DBSCAN) algorithm, we undertake the block adjustment with a bias-compensated rational function model (RFM) aided to improve the geometric performance of these multi-mode GF-3 satellite SAR images. Different weight strategies are proposed to develop the normal equation matrix according to the error sources analysis of GF-3 satellite SAR images, and the preconditioned conjugate gradient (PCG) method is utilized to solve the normal equation. The experimental results indicate that our proposed method can improve the geometric positioning accuracy of GF-3 satellite SAR images within 2 pixels.

## 1. Introduction

The GaoFen-3 (GF-3) satellite is one of the major projects of the the High-Resolution Earth Observation System Project in China [1]. It is the first civilian microwave remote sensing imaging satellite and the first C-band full-polarization synthetic aperture radar (SAR) satellite in China, with the highest resolution up to 1 m. It has many characteristics such as large coverage, long-life operation, high resolution and multiple imaging modes [2], which is helpful to provide remote sensing data with high quality and precision for land and resources monitoring, catastrophic weather warning and so on. There are 12 imaging modes to achieve the image resolution ranging from 1 m to 500 m and the image width ranging from 10 km to 650 km. Since the GF-3 satellite launched on 10 August 2016 by the China Academy of Space Technology (CAST), many researches have been performed on these multi-mode SAR images from the GF-3 satellite. Zhu et al. [3] proposed a method of sidelobe suppression based on the sparsity constraint regularization to reduce sidelobes of GF-3 images in sea areas. In addition, three different restriction principles were proposed as a joint one in [4] to keep the image sharpness while preserving the scattering mechanism as well as speckle reduction of GF-3 images. Moreover, Liu et al. [5] proposed a new method to discriminate water and building shadows with a single SAR image from the GF-3 satellite. However, seldom of these previous studies focused on the improvement of geometric performance of GF-3 satellite SAR images.

Due to the the capability of high-resolution globe imaging of spaceborne SAR [6], researches on spaceborne SAR technology have been carried out in many countries. With the development of spaceborne SAR sensors, the quality of image product has been improved in recent years such as the ALOS PALSAR [7], The COSMO-SkyMed [8] and the TerraSAR-X [9] satellite. The geometric performance of these spaceborne SAR systems can reach the level of less than 10 m. Especially for the TerraSAR-X, the geo-positioning accuracy of its product can reach the order of decimeter level with a precise geometric calibration [10]. In addition, the Chinese GF-3 satellite is similar to the TerraSAR-X satellite with 12 working modes such as spotlight, strip and others. The resolution of these obtained images is different according to the working mode. Therefore, an efficiency method is needed to improve the geometric positioning accuracy for these multi-mode images from the GF-3 satellite.

To improve the geometric performance of multi-mode GF-3 satellite SAR images, block adjustment is an effective approach which can improve the geometric consistency and accuracy [11]. There are four common methods to achieve the block adjustment procedure: direct georeferencing (rigorous sensor model) [12], bundle adjustment [13], the rational function model (RFM) [14] and the Legendre or Fourier polynomial parameters. The first three methods have been compared in [15] and many researches have been done based on them. For example, Cheng et al. [16] proposed a method to improve the geo-positioning of images from TerraSAR based on a rigorous Range-Doppler (RD) model for SAR sensors. Huang and He [17] introduced a method the improve the geometric positioning accuracy of SAR images with ground control points (GCPs) based on the RFM. In addition, a precise geometric correction model and algorithms were developed in [18] to improve the geometric performance of RADASAT-1 SAR images. In addition, the fourth methods were presented by Tang. In 2012, He proposed a new family of rigorous and flexible mathematical self-calibration additional parameters (APs) for airborne camera calibration based on the Laplace’s Equation and the Fourier Theorem [19], and later a new family of Legendre APs is developed in [20]. Both methods are effective to achieve the accuracy improvement and overcome non-detected calibration errors. However, most of these researches need the help of GCPs to improve the geometric positioning accuracy, and the acquisition of GCPs consuming a lot of manpower and finance are always difficult, especially in some areas which are difficult for humans to reach. Moreover, the RFM is the most widely used model in block adjustment considering its simplicity of implementation and standardization [21]. Therefore, we proposed a block adjustment method based on the RFM without GCPs to improve the geometric positioning accuracy of GF-3 satellite SAR images.

Before we undertook the block adjustment procedure, it is of great significance to analyse the data distribution. In addition, the tie point sets acquisition step have a great influence to the results of block adjustment. Well-distributed and accurate tie point sets can contribute to a good convergence and efficiency. Therefore, the forward intersection method is used to extract the coordinates of these tie points considering the characteristics of GF-3 SAR stereo image pairs [22], and the density-based spatial clustering of applications with noise (DBSCAN) [23] method is applied to achieve the analysis of these tie point sets. The DBSCAN method is a widely used data clustering method in the field of statistical learning. Compared with traditional methods such as Decision Trees [24] and K-means [25], the DBSCAN method can automatically cluster the dataset based on the density distribution.

In this paper, we proposed a new method based on the error sources analysis to improve the geometric positioning accuracy of GF-3 satellite SAR images without using GCPs, and the remainder of this paper is organized as follows: In Section 2, we discuss the error sources which influence the geometric positioning accuracy of GF-3 satellite SAR images. Then, the proposed method to undertake the block adjustment procedure is introduced in Section 3 and the details of the experimental dataset are shown in Section 4. Section 5 shows the analysis of experimental results. Finally, discussions and conclusions are presented in Section 6.

## 2. Error Sources of GF-3 Satellite SAR Imagery

According to the imaging principle of SAR sensors, the spatial location of a target point is determined by two aspects: the distance between the target point and radar sensor which is determined by the echo time, and the Doppler characteristic of the target. In addition, three equations are applied to represent the relationships [26]: the SAR range equation, the SAR Doppler equation and the earth model equation. All three equations are shown as Equation (1):

(1) $$\left\{\begin{array}{c} R=|R_{s}-R_{T}|=\frac{c_{\tau }}{2}\\ f_{Dc}=\frac{2}{\lambda_{R}}\left(V_{s}-V_{T}\right)\left(R_{s}-R_{T}\right)\\ \frac{x_{t}^{2}+y_{t}^{2}}{{\left(R_{e}+h\right)}^{2}}+\frac{Z_{t}^{2}}{R_{p}^{2}}=1 \end{array}\right.$$

where *R* is the distance between the target and satellite platform, $R_{S}$ and $R_{T}$ are the position vector of the satellite platform and target, *c* is the light speed and τ represents the time delay; $f_{Dc}$ is the Doppler centroid frequency, $V_{S}$ and $V_{T}$ are the velocity vector of the satellite platform and target; $\left(x_{t},y_{t},z_{t}\right)$ is the target position vector, *h* is the target height relative to the earth model, $R_{e}$ and $R_{p}$ are the equatorial radius and the polar radius of the earth with $R_{p}=\left(1-f\right)\left(R_{e}+h\right)$, and *f* is a flattening factor [27].

According to Equation (1), the geo-positioning accuracy of GF-3 satellite SAR images mainly depends on the echo time delay error, the target elevation error, the ephemeris error of the satellite platform and the doppler center frequency error [28].

### 2.1. Echo Time Delay Error

The position of a target in slant range is determined by the incident angle and the slanting distance between the target and the satellite platform. In addition, the slanting distance is measured by the total time delay between the control signal and the echo signal. Therefore, errors of the time delay of the echo signal will lead to errors in the slant range. The relationship between the distance in slant range and time delay is represented as Equation (2):

(2) $$R=0.5c\left(\tau -\tau_{c}\right)=0.5c\tau_{e}$$

where *R* represents the distance in slant range and $\tau_{e}$ is the time delay between the time of control signal $\tau_{c}$ and the echo signal τ, *c* is the light speed.

The transmission rate of electromagnetic wave passing through the atmosphere is different from that through the ionosphere [29]. The electromagnetic wave was refracted in the ionosphere and the time delay of the echo signal is increased, which is related to the wavelength, the height of the platform and the sunspot activity [30]. As a result, the echo time delay error can result in a slant range error for the geo-positioning accuracy of GF-3 satellite images.

### 2.2. Target Elevation Error

**Figure 1:**
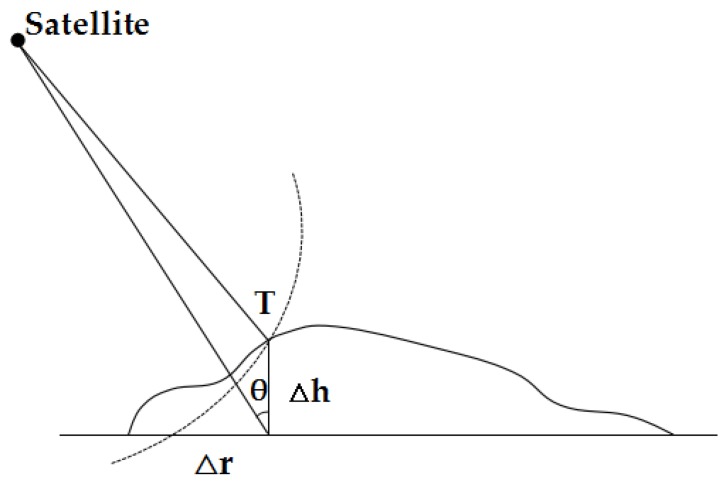
Geo-positioning error caused by the target elevation error.

Considering the side looking imaging characteristics of the GF-3 SAR sensor, we can only obtain the distance and Doppler information. As shown in Figure 1, the slant range error caused by the target elevation error is calculated as Equation (3)

(3) $$\Delta r=\frac{\Delta h}{\tan(\theta )}$$

where Δr and Δh are the slant range positioning error and elevation positioning error, respectively; θ is the incident angle.

Therefore, the slant range positioning error will increase with the decrease of the incident angle. Moreover, the elevation error will lead to an image distortion in the imaging process, which will increase the horizonal positioning error in image-space.

### 2.3. Ephemeris Error

The ephemeris error of the satellite location can be decomposed into 3 components: errors in the along-track direction, errors in the cross-track direction and errors in the radial direction. Location errors in the along-track direction is the main source of errors in azimuth range while it has little influence on errors in slant range. Similarly, errors in the cross-track direction mainly cause errors in slant range while the effect of the Earth’s rotation on the azimuth position can be ignored. Moreover, errors in the radial direction will lead to errors of the incident angle and Doppler shift. Besides, the ephemeris error of the satellite velocity vector can also be separated into the above three directions, and errors in the direction of slant range are small enough to be ignored while the azimuth range error is the main error.

### 2.4. Doppler Center Frequency Error

In the practical running of the satellite platform, there is a certain offset of the Doppler centroid frequency between the calculated value and the actual value due to the attitude error of the satellite platform and errors in the signal processing procedure. In addition, the difference between the Doppler centroid frequency used in azimuth compression and the actual value will lead to an offset in the azimuth range. However, errors of the estimation of the Doppler centroid frequency can be controlled within 3 Hz with the help of the clutter lock and self-focusing technology [31]. Therefore, the azimuth range error caused by the Doppler centroid frequency error can be ignored.

## 3. Methodology

**Figure 2:**
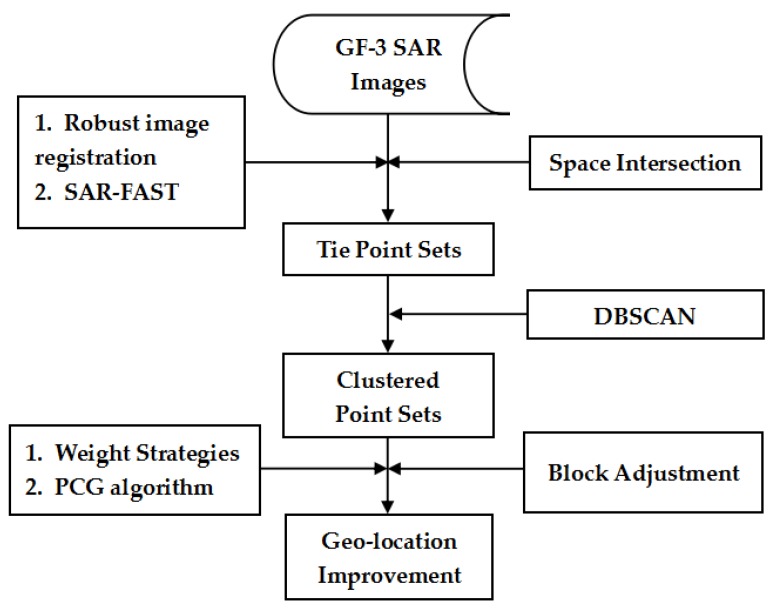
Flow chart of our proposed method.

Figure 2 shows the main steps of our proposed method. Firstly, multi-mode GF-3 satellite SAR images are applied with the help of a robust SAR image registration method [32] and the SAR-features from accelerated segment test (SAR-FAST) corner detection method [33] to obtain the tie point sets. Then, the original object-space coordinates of all tie points are calculated by the space intersection method with the help of GF-3 stereo image pairs. In addition, the DBSCAN algorithm is utilized to separate points with big geo-positioning error from others. With the clustered dataset, we undertake the block adjustment procedure based on the RFM with an affine transformation model to compensate the bias of the rational polynomial coefficients (RPCs). Some weight strategies based on the error sources of GF-3 satellite SAR images are proposed to improve the geometric positioning accuracy. In addition, the preconditioned conjugate gradient (PCG) algorithm is utilized to solve the normal equation matrix defined with the help of RPCs.

### 3.1. Tie Point Sets Acquisition

Firstly, a tie point in image *I* is automatically detected with a corner detection program developed by our team. With the help of RPCs, the original object-space coordinates of this tie point can be obtained, and the same coordinates in image *T* which have the same overlap area in image *I* can be obtained. Due to the bias of RPCs, the calculated image-space coordinates cannot represent the object correctly in image *T*. As a result, a 19 × 19 window around the detected tie point in image *I* are extracted as a standard mask and a 300 × 300 window around the calculated image point in image *T* which represents the same object can be obtained. In addition, the coarse registration step have been done [32].

**Figure 3:**
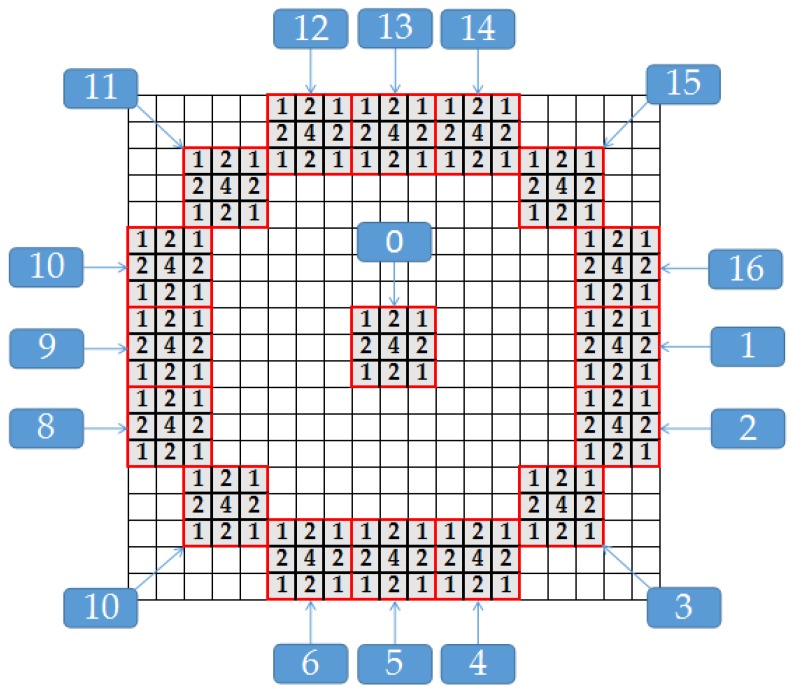
The local processing 19 × 19 window and Gaussian templates.

After the coarse registration, the same 19 × 19 mask are applied in the 300 × 300 window of image *T* to achieve the accurate registration of the tie point. Firstly, the gray-scale transformation is utilized to reduce the influence of radiation difference in different images [34]. Then, the 19 × 19 mask are applied with the SAR-FAST corner detection method which is originated from the traditional Features from Accelerated Segment Test (FAST) corner detection method to search all regions in the 300 × 300 window of image *T*. Considering the effect of speckle noise in SAR images, a 3 × 3 Gaussian template is used in the mask. As shown in Figure 3, the 19 × 19 mask with 3 × 3 templates are applied to search in the 300 × 300 window of image *T*. In addition, the measurement of the similarity are determined as Equation (4).

(4) $$\begin{array}{c} R_{a}\left(k\right)=\frac{\mu (0)}{\mu (k)},\;for\;k=1,2,\ldots 16\\ d_{k}=\left\{\begin{array}{c} 0,\;\mathrm{for}\;1/Th<R_{a}\left(k\right)<Th\\ 1,\;\mathrm{for}\;R_{a}\left(k\right)<Th\\ 2,\;\mathrm{for}\;R_{a}\left(k\right)>1/Th \end{array}\right. \end{array}$$

where μ(0) represents the mean pixel value of the central template 0 in Figure 3, and μ(k) denotes the average of the pixels’ value in template *k*. Th is a threshold value set defined by experience and $d_{k}$ is an evaluation function as shown.

Usually there will be more than one candidate points in image *T*. Therefore, we assign a score function to each candidate point based on the distribution of values based on the ratio in $d_{k}$. The more similarity between the value and distribution of $d_{k}$ in image *T* and image *I*, the higher is its score. With the obtained score, a non-maximal suppression method [35] is utilized to select the best matching point in image *T*. In addition, the tie points can be obtained accurately with these efficiency methods.

### 3.2. Forward Intersection

**Figure 4:**
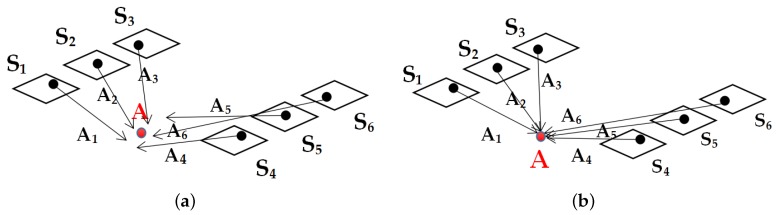
An Example of the space intersection method. $S_{1}$∼$S_{6}$ are different observations of the same object *A* (the red points) at different times, and $A_{1}$∼$A_{6}$ are the calculated corresponding coordinates in object-space with the help of RPCs.

After all tie points are extracted, the coordinates of these tie points are needed to be calculated before the adjustment step. With the help of these GF-3 stereo image pairs, the space forward intersection [22] method is suitable to be applied. The coordinates in object-space which represents the same objection in different images are always distinct due to the bias of the RPCs. As shown in Figure 4a, different observations obtained at different times with different RPCs may cause the difference of the object-space coordinates of the same object. Therefore, overdetermined equations are established based on the multi-observation dataset to reduce the influence of the weak convergence of the object-space coordinates as Equation (5).

(5) $$\left[\begin{array}{ccc} \frac{\partial F_{r}^{0}}{\partial P} & \frac{\partial F_{r}^{0}}{\partial L} & \frac{\partial F_{r}^{0}}{\partial H}\\ \frac{\partial F_{c}^{0}}{\partial P} & \frac{\partial F_{c}^{0}}{\partial L} & \frac{\partial F_{c}^{0}}{\partial H}\\ \vdots & \vdots & \vdots \\ \frac{\partial F_{r}^{n}}{\partial P} & \frac{\partial F_{r}^{n}}{\partial L} & \frac{\partial F_{r}^{n}}{\partial H}\\ \frac{\partial F_{c}^{n}}{\partial P} & \frac{\partial F_{c}^{n}}{\partial L} & \frac{\partial F_{c}^{n}}{\partial H} \end{array}\right]\left[\begin{array}{c} \Delta P\\ \Delta L\\ \Delta H \end{array}\right]=\left[\begin{array}{c} l_{r}^{0}\\ l_{c}^{0}\\ \vdots \\ l_{r}^{n}\\ l_{c}^{n} \end{array}\right]=l$$

where $\left(F_{r}^{n},F_{c}^{n}\right)$ represent the error functions between the calculated and extracted image-space coordinates in the *n*th image; (P,L,H) are the normalized object-space coordinates and (ΔP,ΔL,ΔH) are the corrections of the object-space to be solved; *l* is the vector of residual errors.

(6) $$\left\{\begin{array}{cc} F_{r} & =\frac{Num_{L}\left(P,L,H\right)}{Den_{L}\left(P,L,H\right)}\cdot Line\_Scale+Line\_Off-r\\ F_{c} & =\frac{Num_{S}\left(P,L,H\right)}{Den_{S}\left(P,L,H\right)}\cdot Sample\_Scale+Sample\_Off-c \end{array}\right.$$

Moreover, in Equation (6), (r,c) are the extracted image-space coordinates; Line_Off, Line_Scale, Sample_Off, and Sample_Scale are the scale and translation operators of the calculated image-space coordinates with RPCs, and $Num_{L},Den_{L},Num_{S}$, and $Den_{S}$ are the rational polynomial model determined by 80 RPCs with the degree no more than three [14] as shown in Equation (7):

(7) $$\left\{\begin{array}{c} \frac{Num_{L}\left(P,L,H\right)}{Den_{L}\left(P,L,H\right)}=\frac{a^{T}u}{b^{T}u},\\ \frac{Num_{S}\left(P,L,H\right)}{Den_{S}\left(P,L,H\right)}=\frac{c^{T}u}{d^{T}u}, \end{array}\right.$$

where u=[1 *L* *P* *H* LP LH PH $L^{2}$ $P^{2}$ $H^{2}$ PLH $L^{3}$ $LP^{2}$ $LH^{2}$ $L^{2}P$ $P^{3}$ $PH^{2}$ $L^{2}H$ $P^{2}H$ $H^{3}]$, $a=[a_{0}$ $a_{1}$ … $a_{19}]$, $b=[b_{0}$ $b_{1}$ … $b_{19}]$, $c=[c_{0}$ $c_{1}$ … $c_{19}]$, and $d=[d_{0}$ $d_{1}$ … $d_{19}]$ are the rational polynomial coefficients.

Transforming Equation (5) in the matrix form with an additional condition as Equation (8):

(8) $$\left\{\begin{array}{c} Fx=l,\\ \min\{\sum_{i=1}^{n}x^{2}\}, \end{array}\right.$$

where *F* is the design matrix, *l* is the residual error matrix and *x* is the matrix of unknowns. With the singular value decomposition applied on the designed matrix *F*, the least-squares solutions can be obtained with all observations converge to the same object *A* as shown in Figure 4b.

### 3.3. Data Preprocessing

With the image-space and object-space coordinates obtained in the first two steps, the original error distribution can be obtained. After the forward intersection step, all extracted object-space coordinates share the same value in each tie point sets. However, the calculated object-space coordinates according to the image-space coordinates vary due to the bias of RPCs and inaccuracy in the tie points acquisition step. Therefore, the difference between the calculated and extracted object-space coordinates of all tie points can be distributed together. In addition, the DBSCAN [23] algorithm is applicable to eliminate data with big errors compared with others.

**Figure 5:**
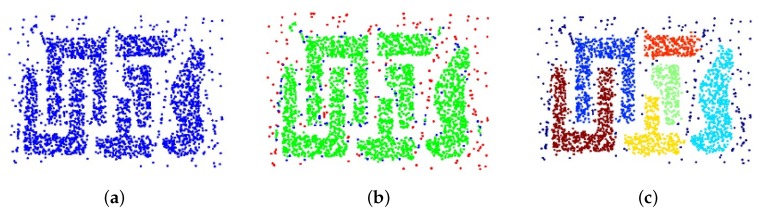
The explanation of the DBSCAN algorithm. (**a**) is the original distribution of the dataset; (**b**) is the distribution of different type of the dataset: core (green) points, border (blue) points and noise (red) points; (**c**) is the cluster results with the DBSCAN algorithm applied to the dataset.

Figure 5 displays an example of the DBSCAN algorithm. Supposing the original dataset is distributed as shown in Figure 5a with different shapes. To get the results in Figure 5c, we divided all data into 3 types: core points (these green points distributed inside the dense regions in Figure 5b), border points (these blue points distributed at the edge of the dense regions in Figure 5b), and noise points (these red points distributed in sparse regions in Figure 5b). Based on the density of the data distribution, 2 parameters are defined—Eps represents the distance between neighborhood points and Minpt is the number of points in a category. Searching randomly from one point in Figure 5a, if there are more than Minpt points within the distance of Eps, these points can form a category. In this way, many categories can be established with an approximate set of these 2 parameters and points do not belong to any category are considered as ‘noise’ points. Compared with traditional clustering method, the DBSCAN method can automatically detect the categories with no bias in shape and noise points can be separated from others with no prior information. Based on the data clustering method, points with big errors can be extracted and eliminated from the dataset.

### 3.4. Free Block Adjustment

With the help of RPCs, the RFM is suitable with a bias-compensated affine transformation model aided to undertake the block adjustment procedure. In addition, the RFM represents the relationship between the image-space and object-space with the follow formula

(9) $$\left\{\begin{array}{cc} X & =\frac{Num_{L}\left(P,L,H\right)}{Den_{L}\left(P,L,H\right)}=\frac{a^{T}u}{b^{T}u}\\ Y & =\frac{Num_{S}\left(P,L,H\right)}{Den_{S}\left(P,L,H\right)}=\frac{c^{T}u}{d^{T}u} \end{array}\right.$$

where (X,Y) is the normalized image-space coordinates, and the meaning of other parameters in Equation (9) are the same as shown in Equation (7).

The RFM is an approximation of the rigorous sensor model, that is the reason of the inaccuracy of the RPCs. Therefore, an affine transformation model is applied to compensate the bias in RPCs as Equation (10):

(10) $$\left\{\begin{array}{cc} \Delta r & =a_{0}+a_{1}\cdot Sample+a_{2}\cdot Line\\ \Delta c & =b_{0}+b_{1}\cdot Sample+b_{2}\cdot Line \end{array}\right.$$

with the de-normalized image-space coordinates (Sample,Line) defined in Equation (11) as

(11) $$\left\{\begin{array}{c} Line=\frac{Num_{L}\left(P,L,H\right)}{Den_{L}\left(P,L,H\right)}\cdot Line\_Scale+Line\_Off\\ Sample=\frac{Num_{S}\left(P,L,H\right)}{Den_{S}\left(P,L,H\right)}\cdot Sample\_Scale+Sample\_Off \end{array}\right.$$

where (Δr,Δc) represent the adjustable functions and $a_{0}$, $a_{1}$, $a_{2}$, $b_{0}$, $b_{1}$ and $b_{2}$ are the adjustment parameters for an image [14]. Based on this, the block adjustment observation equations in image-space is defined as shown in Equation (12)

(12) $$\left\{\begin{array}{cc} G_{r} & =\Delta r+Line-r=a_{0}+a_{1}\cdot Sample+a_{2}\cdot Line+Line-r\\ G_{c} & =\Delta c+Sample-c=b_{0}+b_{1}\cdot Sample+b_{2}\cdot Line+Sample-c \end{array}\right.$$

where $\left(G_{r},G_{c}\right)$ are the error functions and (r,c) are the extracted image-space coordinates. In addition, the matrix form of Equation (12) can be established in the form of Equation (13) as

(13) $$V=At+Bs-l=\left[\begin{array}{cc} A & B \end{array}\right]\left[\begin{array}{c} t\\ s \end{array}\right]-l$$

where *A* and *B* are the designed matrix which consists of the partial derivatives of $\left(F_{r},F_{c}\right)$ to $(a_{0}$, $a_{1}$, $a_{2}$, $b_{0}$, $b_{1}$, $b_{2})$ and (P,L,H); *t* and *s* are the correction vectors of the adjustment parameters and object-space coordinates; *V* is the residual vector and *l* is the difference of the extracted and calculated coordinates in image space. With the Gaussian-Newton model applied to Equation (13), the the normal equation can be rewritten as shown in Equation (14)

(14) $$\left[\begin{array}{cc} A^{T}PA & A^{T}PB\\ B^{T}PA & B^{T}PB \end{array}\right]\left[\begin{array}{c} t\\ s \end{array}\right]=\left[\begin{array}{c} A^{T}Pl\\ B^{T}Pl \end{array}\right],$$

where *P* is the weight matrix, and the simplified normal equation can be written as Equation (15)

(15) $$\left[\begin{array}{cc} U & W^{T}\\ W^{T} & V \end{array}\right]\left[\begin{array}{c} t\\ s \end{array}\right]=\left[\begin{array}{c} l_{u}\\ l_{v} \end{array}\right].$$

and the results of Equation (15) can be obtained with the help of Gaussian elimination as shown in Equation (16)

(16) $$\left\{\begin{array}{c} \left(U-WV^{-1}W^{T}\right)\cdot t=l_{u}-WV^{-1}l_{v},\\ V\cdot s=l_{v}-W^{T}t. \end{array}\right.$$

### 3.5. Weight Strategy

In the above free block adjustment, the unknowns are divided into two groups: the adjustable parameters and the object-space coordinates. Due to the lack of control points, the weights strategy plays an important role in the free block adjustment step. From the previous study in [36], we assume that the adjustable parameters in *t* of the same image share the same value while the errors of the object-space coordinates in *s* are independent from each other. According to the error sources of GF-3 satellite SAR imagery in Section 2, errors in the slant range are much bigger than errors in the azimuth range and elevation errors, and errors in the slant range are in connected with the elevation errors. However, with the limitation of the product level of the GF-3 satellite images, sources cause the errors are mainly the incident angle, the image resolution and the orbit. Therefore, weights of *s* and *t* are updated during the iteration as Equation (17)

(17) $$\begin{array}{c} \left\{\begin{array}{c} P_{ss_{i,j}}^{m}=\frac{l_{j}}{l_{sum}^{i}}\cdot \frac{1}{q_{j}}\cdot \frac{tan\theta_{j}}{E_{j,error}^{i}}\\ P_{sa_{i,j}}^{m}=\frac{l_{j}}{l_{sum}^{i}}\cdot \frac{1}{q_{j}}\cdot \frac{1}{A_{j,error}^{i}}\\ P_{se_{i,j}}^{m}=\frac{l_{j}}{l_{sum}^{i}}\cdot \frac{1}{q_{j}}\cdot \frac{1}{E_{j,error}^{i}} \end{array}\right.\\ P_{t_{i,j}}^{m}=\left\{\begin{array}{cc} \frac{l_{j}}{l_{sum}^{i}}\cdot \frac{1}{q_{j}}\cdot \frac{k^{j}}{\sigma_{j}^{m-1}\sum_{n=1}^{k^{j}}d_{n}}, & \mathrm{for}\;a_{0}^{j},b_{0}^{j}\\ \frac{l_{j}}{l_{sum}^{i}}\cdot \frac{1}{q_{j}}\cdot \frac{k^{j}}{\sigma_{j}^{m-1}\sum_{n=1}^{k^{j}}d_{n}}/H_{j}, & \mathrm{for}\;a_{1}^{j},b_{1}^{j}\\ \frac{l_{j}}{l_{sum}^{i}}\cdot \frac{1}{q_{j}}\cdot \frac{k^{j}}{\sigma_{j}^{m-1}\sum_{n=1}^{k^{j}}d_{n}}/W_{j}, & \mathrm{for}\;a_{2}^{j},b_{2}^{j} \end{array}\right. \end{array}$$

where $P_{ss_{i,j}}^{m}$, $P_{sa_{i,j}}^{m}$ and $P_{se_{i,j}}^{m}$ represent the weights of object-space coordinates of the *i*-th tie point set in the *j*-th image in the direction of slant range, azimuth range and elevation during the *m*-th iteration; $l_{sum}^{i}$ are the total number of orbit in the *i*-th tie point set including the ascending orbit and descending orbit, and $l_{j}$ is the number of a current class which the *j*-th image belongs to; $q_{j}$ is the resolution of the *j*-th image, and $\theta_{j}$ is the incident angle; $E_{j,error}^{i}$ and $A_{j,error}^{i}$ are the residual elevation error and azimuth range error of the *i*-th tie point set in the *j*-th image. $P_{t_{i,j}}^{m}$ are the weights of the adjustable parameters of the *i*-th tie point set in the *j*-th image during the *m*-th iteration. $d_{n}$ are the image coordinate errors of the *n*-th point in the *j*-th image, and $k^{j}$ is the number of tie points in the *j*-th image; $\sigma_{j}^{m-1}$ is the standard deviation of the difference of all tie points between the calculated coordinates in the iteration step and coordinates extracted in the space intersection step in the *j*-th image; $\left(H_{j},W_{j}\right)$ is the length and width of the *j*-th image.

In our experiment, we use four different weight strategies applied on the test dataset. The first one is a identity weight matrix applied on both the object-space coordinates and adjustment parameters. Secondly, we apply $P_{ss_{i,j}}^{m}$, $P_{sa_{i,j}}^{m}$ and $P_{se_{i,j}}^{m}$ to the object-space coordinates and a identity weight matrix to the adjustment parameters. Thirdly, the $P_{t_{i,j}}^{m}$ are applied to the adjustment parameters and an identity weight matrix is utilized to the object-space coordinates. $P_{ss_{i,j}}^{m}$, $P_{sa_{i,j}}^{m}$ , $P_{se_{i,j}}^{m}$ and $P_{t_{i,j}}^{m}$ are utilized together as the 4-th weight strategy. Based on these weight strategies, the influence of the incident angle, the image resolution and orbit can be reduced to a certain extent by comparison.

### 3.6. PCG Algorithm

In the previous study, solutions to solve the rank-deficient normal equation haves seldom been detailed. Traditional methods such as spectrum correction (SC) and conjugate gradients (CG) Method are time-consumed and inefficient. Therefore, we choose the PCG method to achieve the work. Considering a single linear equation as shown in Equation (18)

(18) Ax=B

where *A* is the design matrix, *B* is the residual error vector and *x* is the vector of unknowns.

Compared with the traditional CG method, the PCG method multiplies a preconditioner $M^{-1}$ to the normal equation in order to decrease the condition. There are many kinds of preconditioner which can be applied during the process, such as the block Jacobi preconditioner [37], the QR (where *Q* represents an orthogonal matrix and *R* represents an upper triangular matrix) factorization preconditioner [38] and multiscale preconditioner [39]. In our experiments, we use the block Jacobi preconditioner $M^{-1}$ due to its simplicity and Equation (18) can be written as Equation (19)

(19) $$M^{-1}Ax=M^{-1}B⟺A^{\prime }x=B^{\prime }$$

where $A^{\prime }=M^{-1}A$ and $B^{\prime }=M^{-1}B$. In addition, the iteration step of the PCG method is demonstrated as follows:

(1) Initialization: $x_{0}$ = 0, $r_{0}=B^{\prime }-A^{\prime }x_{0}$, $z_{0}=r_{0}$, k=0.
(2) Iteration:
  (20) $$\begin{array}{c} x_{k+1}=x_{k}+\frac{\left[r_{k},z_{k}\right]}{\left[A^{\prime }z_{k},z_{k}\right]}z_{k}\\ r_{k+1}=B^{\prime }-A^{\prime }x_{k+1}\\ z_{k+1}=r_{k+1}-\frac{\left[r_{k+1},A^{\prime }z_{k}\right]}{\left[z_{k},A^{\prime }z_{k}\right]}z_{k}=r_{k+1}+\frac{{∥r_{k+1}∥}^{2}}{{∥r_{k}∥}^{2}}z_{k}.\\ k=k+1 \end{array}$$
(3) Estimation: if $r_{k}=0$ , or $r_{k}\neq 0$ and $[Az_{k},z_{k}]=0$, $x_{k}$ is the expected result; else return to the iteration step as shown in Equation (20).

## 4. Experimental Dataset

**Figure 6:**
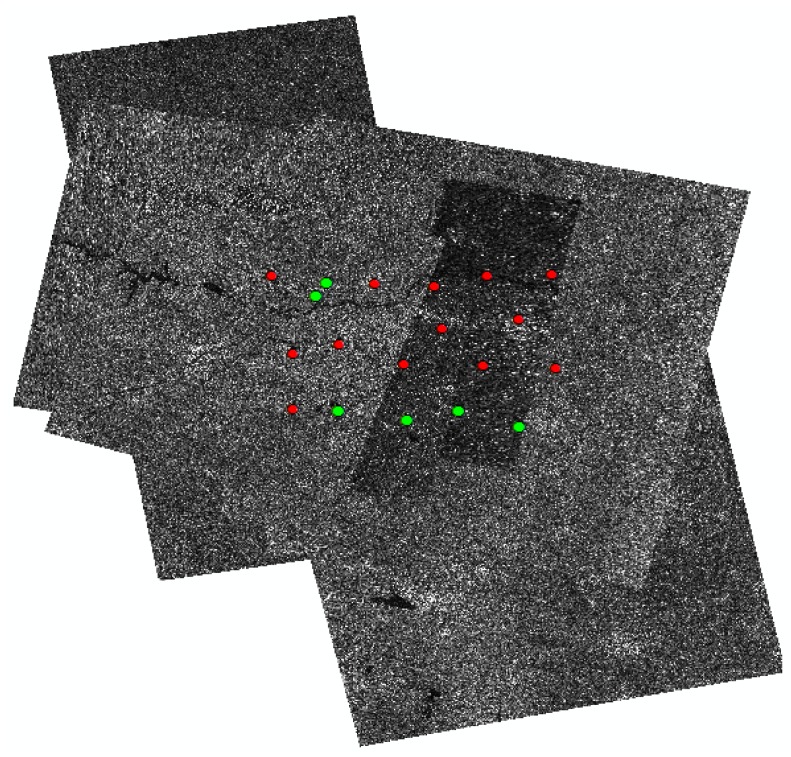
Experimental dataset distribution in Songshan area.

Figure 6 shows the distribution of the test dataset. In our study, a total of 52 multi-mode images from the GF-3 SAR satellite are applied to undertake the block adjustment procedure. In addition, all images are provided with their corresponding RPCs data. The test dataset are acquired from June 2016 to June 2017 covering a total area of 75,000 km${}^{2}$ in Songshan area . There are 19 tie point sets distributed in the experimental area, 6 of which are check point sets (points in green) and a total of 192 tie points including 72 check points (CKPs) are extracted in all images. The maximum number of overlapping images was 19 with most mountains distributed throughout the area. A total of 5 types GF-3 L1A level SAR images are used in our experiments, and the information of the test dataset are listed in Table 1.

Usually, the size of a GF-3 SAR image is about 50 km × 50 km covering a large area, which indicates that the incident angles at perigee and apogee of each image are different. Therefore, the central look angle is utilized to replace the incident angle of each image in our experiment.

## 5. Experimental Results and Analysis

### 5.1. Tie Point Sets Acquisition

**Figure 7:**
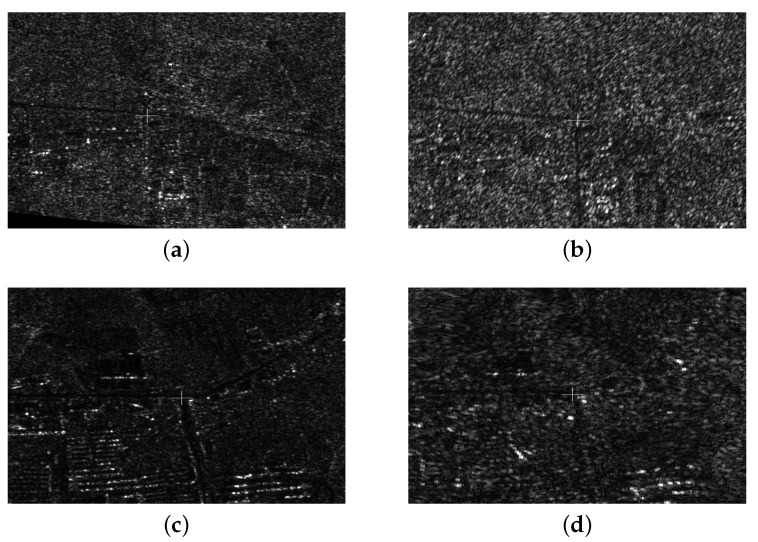
Examples of extracted tie point sets. (**a**,**b**) are two examples of the extracted image-space points from different images in tie point set P17 and (**c**,**d**) are two examples of the extracted image-space point from different images in tie point set P21, where the centers of the crosses are the extracted image-space point correlated with the object in object-space.

Based on the robust registration method, different images can achieve the coarse registration with the help of RPCs. Considering the bias of the RPCs, we applied the SAR-FAST corner detection method to search the candidate tie point in a 300×300 window around the pints calculated based on RPCs. Moreover, 3 × 3 Gaussian templates are added on the 19 × 19 mask in order to reduce the influence of speckle noise. In addition, a non-maximum suppression method is utilized to ensure the best matching point in the tie point sets. Figure 7 gives some examples of tie points in different images.

### 5.2. Free Block Adjustment

With the dataset extracted after data preprocessing, the free block adjustment is conducted with an affine transformation model to compensate the bias of RPCs. Based on the analysis of error sources of GF-3 satellite SAR imagery, we applied different weight strategies on the test dataset to confirm the accuracy of our analysis. After the data preprocessing step, most points with big error have been removed from the dataset which indicates the clustered dataset are dependable. With the PCG algorithm utilized to solve the designed normal equation, we use the root mean square error (RMSE) of the absolute errors of these check points to verify the geometric accuracy in each direction as Equation (21)

(21) $$\left\{\begin{array}{cc} \mu_{X} & =\sqrt{\frac{{\left(X_{c}-X\right)}^{2}}{n}}\\ \mu_{Y} & =\sqrt{\frac{{\left(Y_{c}-Y\right)}^{2}}{n}}\\ \mu_{P} & =\sqrt{\frac{{\left(X_{c}-X\right)}^{2}+{\left(Y_{c}-Y\right)}^{2}}{n}}\\ \mu_{H} & =\sqrt{\frac{{\left(H_{c}-H\right)}^{2}}{n}} \end{array}\right.$$

where $\left(X_{c},Y_{c},H_{c}\right)$ and (X,Y,H) represent the calculated and actual object-space coordinates of check points in latitude, longitude and height, and $\left(\mu_{X},\mu_{Y},\mu_{P},\mu_{H}\right)$ are used to represent the RMSE in latitude, longitude, the resulting vector directions and height.

With the help of RPCs and the resolution of each GF-3 SAR image, we can convert these errors in the direction of slant range and azimuth range into errors in the direction of latitude and longitude. Therefore, we proposed 4 different weight strategies to confirm our analysis of the error sources of GF-3 satellite SAR images. Weight strategy 1 represents a unity matrix applied in the process of block adjustment. Weight strategy 2 represents the case that $P_{t_{i,j}}^{m}$ applied on the affine transformation parameters and weight strategy 3 represents the case that $P_{s_{i,j}}^{m}$ (including $P_{ss_{i,j}}^{m}$, $P_{sa_{i,j}}^{m}$ and $P_{se_{i,j}}^{m}$ ) applied on the object-space coordinates. Weight strategy 4 represents the combination of weight strategy 2 and 3.

**Figure 8:**
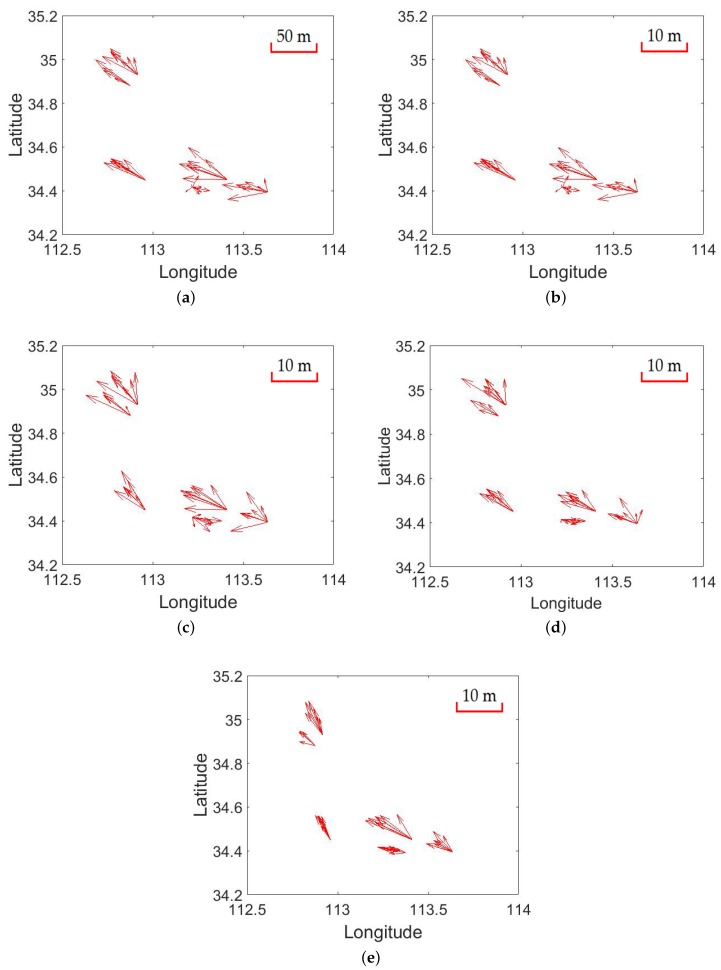
Error distribution of the test dataset with different weight strategies. (**a**) is the error distribution before block adjustment; (**b**) is the error distribution after the block adjustment procedure with weight strategy 1; (**c**) is the error distribution after the block adjustment procedure with weight strategy 2; (**d**) is the error distribution after the block adjustment procedure with weight strategy 3 and (**e**) is the error distribution after the block adjustment procedure with weight strategy 4.

With different weight strategies utilized on the same dataset, the residual error distributions before and after block adjustment are displayed in Figure 8. From the figure, we can see that the convergence of the error distribution after block adjustment is better than that before block adjustment. Moreover, the value of the errors reduced a lot after block adjustment and they converged to almost the same direction, which indicates the efficiency of the block adjustment method. As for the numeric analysis, the value of these residual errors after block adjustment are quite smaller than that before block adjustment, and these proposed additional weight strategies make the results better than a identity weight matrix while comparing the results in Figure 8b–e. Table 2 shows the numeric value of these results. The standard deviation (STD) and RMSE are calculated as shown in Equation (22)

(22) $$\begin{array}{c} X_{RMSE}=\sqrt{\frac{\sum_{t=1}^{N}X_{i}^{2}}{N}},\\ X_{STD}=\sqrt{\frac{\sum_{t=1}^{N}{\left(X_{i}-\mu_{X}\right)}^{2}}{N}}, \end{array}$$

where *N* is the number of points in each check point set; $X_{i}$ is the residual error of the *i*th check point in the resulting vector directions of latitude and longitude, and $\mu_{X}$ is the mean value of all $X_{i}$ in the test check point set.

**Figure 9:**
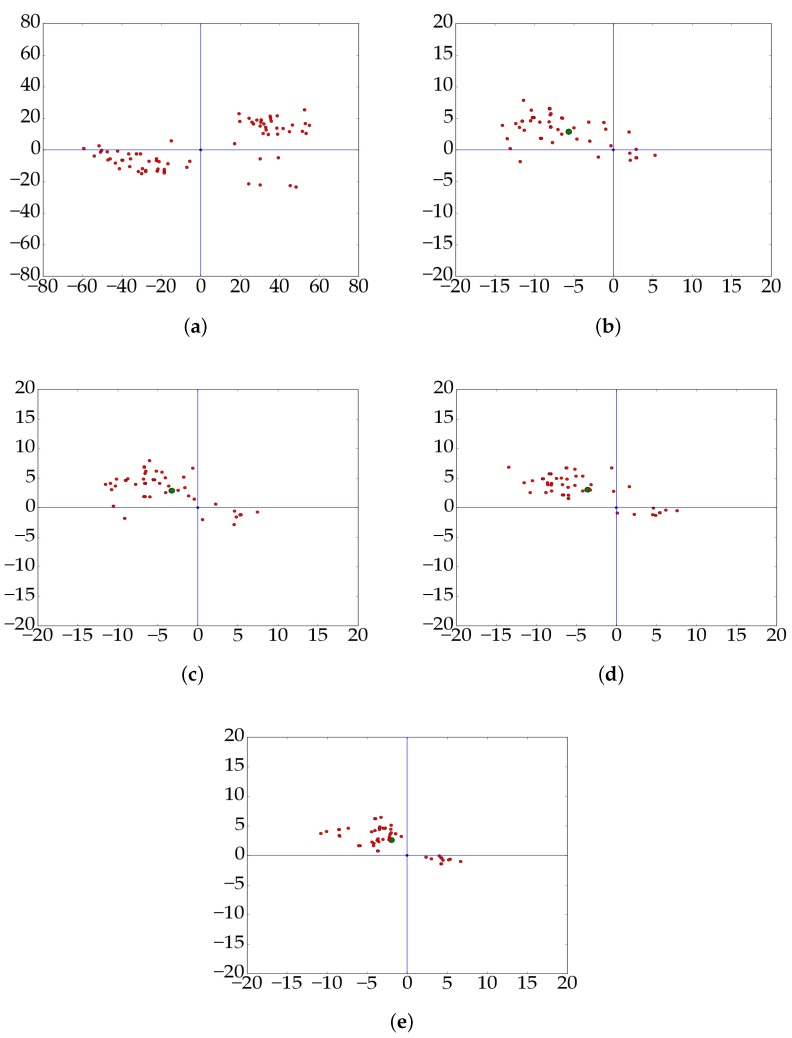
Error distribution of all check points before and after block adjustment. (**a**) is all check points’ error distribution before block adjustment; (**b**) is all check points’ error distribution after the block adjustment procedure with weight strategy 1; (**c**) is all check points’ error distribution after the block adjustment procedure with weight strategy 2; (**d**) is all check points’ error distribution after the block adjustment procedure with weight strategy 3 and (**e**) is all check points’ error distribution after the block adjustment procedure with weight strategy 4.

**Figure 10:**
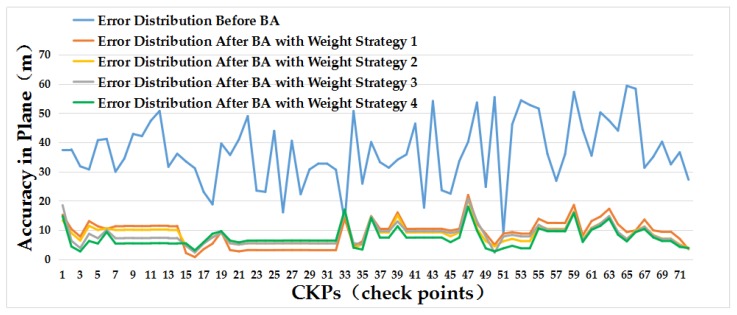
Plane error distribution of our test dataset. BA is the abbreviation of free block adjustment.

While comparing the results in Figure 8a,b, we can see that errors in the direction of longitude are bigger than that in the direction of latitude, which can be reflected to errors in the direction of slant range and azimuth range by the incident angle. Therefore, we proposed different weight strategies to reduce these errors caused by the above analysis. The RMSE of these residual errors in the direction of longitude are bigger than that in the direction of latitude after block adjustment with an identity weight matrix (weight strategy 1), while the difference of residual errors in different directions have been reduced largely after block adjustment with our proposed weight strategy 4. Moreover, the residual error distributions of all check points can confirm the validity of our proposed weight strategies based on the error sources analysis as shown in Figure 9. The red points are residual errors of all check points and the green point is the cluster center of all error points. Compared results from Figure 9b–e, we can see an obvious convergence of the residual errors in the direction of longitude and latitude with different weight strategies with the results of weight strategy 4 converge best, which indicates the efficiency of our proposed error sources-based weight strategies. The same conclusion can be obtained with the help of Figure 10. The blue line represents the error distribution of all check points before block adjustment. The orange line is the residual error distribution after block adjustment with weight strategy 1. The yellow line is the residual error distribution after block adjustment with our proposed weight strategy 2. The gray line is the residual error distribution after block adjustment with weight strategy 3 and the green line is the residual error distribution after block adjustment with weight strategy 4.

## 6. Conclusions

In this paper, we put forward an error sources-based method to improve the geometric positioning accuracy of GF-3 satellite SAR images. By analysing the error sources of SAR images from the GF-3 satellite, we proposed different weight strategies to reduce errors mainly in slant range, azimuth range and height. Dataset of Songshan area were test in our experiment. Based on the DBSCAN algorithm, we clustered all data to eliminate points with big errors aiming to enhance he credibility of the selected dataset first. With the clustered dataset, we conduct the block adjustment with an affine transformation model aided to compensate the bias of RPCs. Moreover, different efficient weight strategies and the PCG algorithm were applied to develop the normal equation matrix and solve it. The experimental results showed that the RMSE of residual errors converged to 10.53 m in plane and 7.73 m in height after block adjustment with an identity weight matrix compared the results 40.00 m in plane and 19.53 m in height before block adjustment, which indicates the efficiency of the block adjustment model. In addition, the geometric positioning accuracy improved with the help of our proposed weight strategies, especially it improved to 7.99 m in plane and 6.75 m in height with weight strategy 4 which confirms the credibility of our proposed weight strategies based on the error sources analysis. Therefore, the results show a great improvement in geometric positioning accuracy of GF-3 satellite SAR images based on our proposed method, and the residual errors can be controlled within 2 pixels. In addition, we believe it can obtain better results with the increase of image resolution. Further research will be continued with more high-resolution GF-3 SAR images added to achieve a better promotion.

## Figures and Tables

**Table 1:** Information of GF-3 dataset in our experiments.

Imaging Mode	Incident Angle (${}^{\circ }$)	Resolution (m)	Orbit	Number
Fine Strip I (FSI)	19∼50	5	ASC	6
			DEC	17
Fine Strip II (FSII)	19∼50	10	ASC	0
			DEC	2
Standard Strip (SS)	17∼50	25	ASC	4
			DEC	2
Full Polarized Strip I (QPSI)	20∼41	8	ASC	8
			DEC	3
Full Polarized Strip II (QPSII)	20∼38	25	ASC	7
			DEC	3

**Table 2:** Comparison of positioning accuracy with different weight strategies. (BA is the abbreviation of free block adjustment. RMSE represents the root mean square error, STD represents the standard deviation, MAX and MIN are the abbreviation of maximum and minimum, and Lat and Long is the abbreviation of latitude and longitude.)

Item		Max	Min	RMSE	STD
Error Before BA (m)	Long	55.09	−59.34	34.80	36.35
	Lat	25.42	−23.44	12.65	13.32
	Plan	59.35	9.04	40.00	11.28
	Height	27.67	14.94	19.53	2.22
Error After BA Weight Strategy 1 (m)	Long	5.26	−14.07	9.40	5.59
	Lat	7.87	−1.83	4.75	2.81
	Plan	21.99	0.75	10.53	4.44
	Height	12.07	−1.51	7.73	1.58
Error After BA Weight Strategy 2 (m)	Long	7.44	−11.55	7.79	5.47
	Lat	7.99	−2.88	4.88	3.00
	Plan	20.72	2.29	9.19	3.51
	Height	11.30	−0.95	7.22	1.31
Error After BA Weight Strategy 3 (m)	Long	7.75	−13.45	7.89	5.75
	Lat	6.84	−1.28	4.85	2.80
	Plan	20.50	2.51	9.26	3.36
	Height	12.01	−1.70	7.54	1.79
Error After BA Weight Strategy 4 (m)	Long	6.66	−10.79	6.66	4.27
	Lat	6.45	−1.39	4.41	2.46
	Plan	17.99	2.68	7.99	3.27
	Height	9.81	−2.80	6.75	1.17
